# Taking language science to zoom school: Virtual outreach to elementary school students

**DOI:** 10.1111/lnc3.12471

**Published:** 2022-09-11

**Authors:** Kathleen E. Oppenheimer, Lauren K. Salig, Craig A. Thorburn, Erika L. Exton

**Affiliations:** ^1^ Department of Hearing and Speech Sciences University of Maryland College Park Maryland USA; ^2^ Department of Psychology University of Maryland College Park Maryland USA; ^3^ Program in Neuroscience and Cognitive Sciences University of Maryland College Park Maryland USA; ^4^ Department of Linguistics University of Maryland College Park Maryland USA; ^5^ Institute for Advanced Computer Studies University of Maryland College Park Maryland USA

## Abstract

We describe guest speaker presentations that we developed to bring language science to elementary school students via videoconference. By using virtual backgrounds and guided discovery learning, we effectively engage children as young as 7 years in in‐depth explorations of language science concepts. We share the core principles that guide our presentations and describe two of our outreach activities, *Speech Detectives* and *Bilingual Barnyard*. We report brief survey data from 157 elementary school students showing that they find our presentations interesting and educational. While our pivot to virtual outreach was motivated by the Covid‐19 pandemic, it allows us to reach geographically diverse audiences, and we suggest that virtual guest speaker presentations will remain a viable and effective method of public outreach.

## 1 Introduction

When Covid‐19 was declared a pandemic in March 2020, schools closed and in‐person science fairs were cancelled. This posed a challenge for language science faculty and students involved in community outreach that relied on in‐person interactions, such as camps and summer programs (Clark & Trousdale, 2012; Farris‐Trimble & Reid, 2019; McGory & Barlew, 2020), inviting students to college campuses (Lidz & Kronrod, 2014), visiting schools (Denham, 2007; McKee et al., 2015), or staffing booths at science fairs (McKee et al., 2015) and museums (Wagner et al., 2015). We are a group of PhD students who paused our outreach efforts at the start of the pandemic and realized that we would need to adapt our outreach approaches to be done virtually when it became clear that the pandemic had created more than a short‐term disruption.

We took inspiration from teachers and paediatric speech‐language pathologists, who early in the pandemic adapted to the needs of virtual instruction by using interactive virtual backgrounds (Murison & Jeffrey, 2020; PlaySpark, 2020; Russell, 2021). Performing in front of a felt green screen creates a crisper image than simply using a virtual background and facilitates special effects to make presentations more engaging. We expanded the short demonstrations we previously used at booths at in‐person science fairs to create longer, more in‐depth interactive activities that we could present virtually. We engaged students as young as second grade in 45‐min sessions to explore topics from introductory linguistics courses, such as phonological processes and categorical perception. Because language science often does not require special equipment or materials, we found that it is well‐suited to virtual teaching, where students are able to follow along from home and do language science with us, rather than watching us demonstrate a task. This new way of doing outreach required considerably more planning, rehearsal, and teaching skill than shorter booth‐style demonstrations, but also had a greater payoff by allowing students to develop deeper insights into scientific approaches to studying language.

While a great deal of language science outreach research has focused on high school students and general audiences, we are not the first to target elementary students. Denham (2007) taught a linguistics class at a private elementary school, and Farris‐Trimble and Reid (2019) have run day‐long camps for elementary school students. In addition, methods of outreach that involve short, high‐interest demonstrations at museums (Wagner et al., 2015) or science fairs (McKee et al., 2015) are likely to involve students in this age range even if they are not the sole age group targeted. Language science graduate students and faculty at our university previously participated in a range of outreach activities; those that targeted elementary students before the pandemic were primarily booth‐style events where language scientists provided short demonstrations at multi‐age events or at elementary school science fairs.

Bringing language science to elementary school students has many benefits. Language science is relevant to the common core standards for literacy (Denham, 2015; Mulder, 2011) and to science and math curricula (Honda & O'Neil, 1993; Mallinson & Charity Hudley, 2014). Elementary school students studying language science explore different languages and ways of using languages, hopefully before they develop misconceptions about language (O'Neil, 1998). Additionally, students often hold narrow views of what science is and who does it (Chambers, 1983; Finson et al., 1995; Hillman et al., 2014); visits from language scientists may expand students' views of STEM (Science, Technology, Engineering, and Math) careers when they experience a different way of being a scientist by applying the scientific method to language. Our focus on the scientific method by testing hypotheses and gathering data to support a claim promotes scientific literacy in school‐age children (Sandoval et al., 2014). Language science fields are often ‘discovery majors’, meaning that most students do not discover them until college, and we hope to increase awareness of the discipline through outreach to younger students. Our observations coupled with short survey responses from elementary school students suggest that virtual outreach maintains many of these benefits while also allowing us to reach students who are not located near a university with a language science outreach programme.

In this article, we discuss a novel method of language science outreach to elementary school students that we developed and refined during the pandemic. We use a guest speaker approach with engaging lessons that we teach during single visits to classrooms. We describe our methods for engaging classrooms virtually, our guiding principles, and the results of a survey that students complete after our virtual classroom visits. We use the broader term ‘language science’ rather than linguistics to reflect that scientists who study language may come from a variety of academic backgrounds, including psychology, cognitive science, neuroscience, speech‐language pathology, and linguistics.

## 2 OVERVIEW OF OUTREACH VISITS

We developed engaging, interactive language science demonstrations for elementary students that were delivered live over Zoom. We started by adapting the short outreach demonstrations we previously used at in‐person science fairs to create longer presentations that could fill a class period. We strove to maintain the interactive spirit of our in‐person activities while adapting to the large‐group guest speaker events where our virtual outreach takes place. Without a physical booth or one‐on‐one interactions, we had to re‐engineer our demonstrations to keep them engaging and interactive. Green screens were an excellent solution to this problem.

### 2.1 Green screens

**FIGURE 1:**
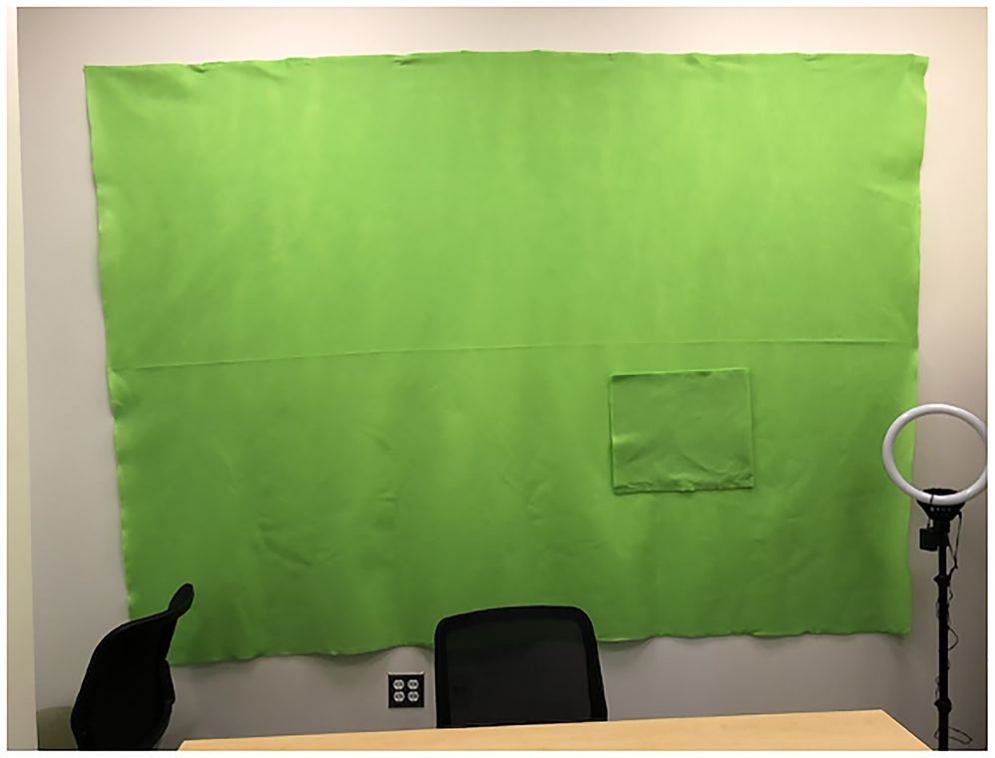
Image of a felt green screen measuring 6 feet by 6 feet. Note the felt pocket that is not visible when a virtual background is used and that allows presenters to hide objects and make them ‘appear’

A green screen is simply a large piece of green felt or cloth used as a backdrop that facilitates the use of virtual backgrounds on videochat (Figure 1). By using felt green screens, we can create fun and useful effects. In some of our demonstrations, we place green felt pockets onto the green screen: items hidden in the pockets beforehand can seemingly appear out of thin air when we pull them out of the pocket during the activity. In most of our demonstrations, we interact with our backgrounds by printing out words or images and gluing them to green felt. The felt pieces stick to the felt background, allowing us to move them around during the presentation. We use these felt pieces to keep running lists of observations that the students have discovered and to introduce hypotheses as students generate them, rather than being tied to a predetermined order of slides. To use slideshows as our virtual backgrounds, we export our slides as images and upload these to our video conferencing software as individual virtual background images so that we appear in our slides.

Much research supports the idea that visuals and interactivity are helpful for learning (e.g., Beauchamp & Kennewell, 2010; Höffler & Leutner, 2007). Anecdotally, we found that students are excited to take part in an activity with fun visuals where their participation directly impacts how the activity proceeds. We intentionally chose clip art from a set representing diverse backgrounds and family structures so that students can see themselves in our demonstrations (Illustrate Your Voice, 2021).

### 2.2 Visit procedure

Each of our demonstrations goes through a piloting and editing process through which we get feedback from peers and from students in our target age range if possible. Once a demonstration is ready, we add it to our repertoire of activities that we can offer to teachers. We connect with teachers by reaching out to local elementary schools, personal connections, and local parent listservs or Facebook groups, and by advertising on our Instagram page.

Once invited to a classroom, we set up our own Zoom meeting with carefully set controls. On the day of our visit, we log onto Zoom, introduce ourselves to the class as language scientists, and begin our activity by asking students to tell us what a hypothesis is. This helps us tailor the rest of the presentation to their current understanding of the scientific method and sets up our focus on forming and modifying hypotheses to explain data. Opening with a question lets us establish how interaction will work throughout our demonstration, which can vary based on teachers' preferences. If all students and presenters are online on their own devices, we encourage the use of the hand‐raise feature in Zoom, the chat feature, and in some specific cases, the reactions feature. In hybrid situations where students are together in‐person but presenters are virtual, teachers often facilitate interaction for us—asking students to raise hands, calling on them, and relaying answers to us on Zoom. Having set the stage for our interactive demonstration, we begin our activity for the day, always keeping in mind our guiding principles for how to lead outreach demonstrations.

## 3 GUIDING PRINCIPLES

In the process of creating and piloting our virtual demonstrations, we found that employing certain strategies or priorities increased student engagement and helped students reach the conclusions that we wanted them to come away with. We use these four guiding principles as goalposts when creating and leading our demonstrations.

### 3.1 Invite problem solving

Rather than simply telling students something about language science, we allow them to discover principles of language science on their own through our scaffolded activities. Students are asked to make hypotheses, test them against provided data, and reformulate hypotheses in an interactive scientific process. This practice aligns with recent science education guidelines to move away from teaching students facts and toward *discovering* core scientific concepts (Loyens et al., 2015; Next Generation Science Standards, 2013). Our methods are most similar to what Alfieri et al. (2011) classified as ‘guided discovery’, where students receive scaffolding from an instructor to figure out scientific principles from a set of materials. Our virtual activities allowed us to shift toward this more rewarding approach as we have time with students to contextualize information and walk them through a discovery process.

### 3.2 Simplify terms

Our activities ask students as young as 7 years old to engage with data sets similar to those we might ask first‐year university linguistics students to work with. To facilitate their learning, we simplify technical terminology, so students can focus on the relevant material. For example, in our *Speech Detectives* activity, students learn about how speech sounds are made. To teach the concept of ‘place of articulation’, we use phrases like ‘How does your mouth feel?’ or ‘Where is your tongue?’ When it is necessary to introduce a new term, we give thorough explanations and repeat a short definition throughout the activity, such as defining ‘voiced’ and ‘voiceless’ sounds as those made by turning the voicebox ‘on’ or ‘off’. This approach allows us to focus on the scientific aspects of the activity without requiring time devoted purely to vocabulary.

### 3.3 Address misconceptions

The general public has their own ideas about language—endorsing various language myths (e.g., about bilingualism, speech disorders, and African American Language) to different degrees (Wagner et al., 2022; Wiley & De Klerk, 2010). As language scientists doing outreach, we want to prevent misconceptions about language. Direct refutation of myths has been shown to be ineffective at best and counterproductive at worst (e.g., Ferrero et al., 2020; Peter & Koch, 2016); instead, we provide data and examples that would lead the audience to arrive at a linguistically sound conclusion that runs counter to common misconceptions. When we create new activities, we begin with extensive brainstorming to determine the take‐away message we want students to get. The takeaway message is usually selected to contradict a common myth, and stating it clearly at the outset helps us focus the rest of the demonstration on leading to that conclusion.

Additionally, as we create activities, we carefully consider if and how students might come away with a take‐away message we did not intend. Could students walk away from a discussion of cross‐linguistic variation in syllable structure thinking that Japanese is a ‘simpler’ language than English? Could they walk away from a presentation about code‐switching thinking that bilingual speakers are confused? If so, we take time to rework our activity to ensure that the information we present does not lead to these conclusions and that we are prepared to appropriately answer any questions that may arise.

Take, for example, our *Bilingual Barnyard* activity, where we compare the phonology of English, German, and Japanese. We are careful to identify characteristics that each language has that the others do not to avoid implying that one language is ‘simple’ or ‘missing’ certain sounds. Another example of this is our use of the term ‘patterns’, rather than ‘rules’, in all of our presentations. While linguists, include undergraduate linguistics students, have a descriptive understanding of the word ‘rule’ (such as in the context of a systematic sound change), elementary school students may interpret ‘rule’ to have a prescriptive meaning, which could reinforce misconceptions about linguistic diversity that we aim to disrupt. To address any misconceptions that do arise, we make sure that the scientist leading the activity has expertise in the topic and in communicating with children, so they can answer questions on the fly. For example, our *Speech Detectives* activity is led by a graduate student who also has training in speech‐language pathology.

### 3.4 Encourage interaction

Our guided discovery approach to outreach requires that students engage with our activities. *They* are the ones forming and testing hypotheses. When creating and testing activities, we plan where we will pause for student contributions and how student responses will change what we do next. Our green screen set‐up with movable felt pieces allows us to choose the order of our slides to address whatever students bring up. Additionally, we offer students multiple ways to interact when possible. When all students are on Zoom, some prefer to respond in the chat or with reaction icons, while others are more than happy to raise their hand and respond verbally.

## 4 CASE STUDIES

In this section, we describe two of our activities and how we adapted them into interactive virtual presentations. Other activities include demonstrations on: categorical perception, bilingual code‐switching, the Stroop task, and sound illusions. Our presentations, along with their intended takeaways, are listed in Table 1.

**TABLE 1:** List of outreach presentations

Activity	Length	Take‐away message	Concepts taught
Bilingual Barnyard	20–45 min	Different languages have different sounds, and patterns for how they combine them.	Phonology and phonotactics; Onomatopoeia
Bilingual Code‐switching (How do bilinguals choose what language to use?)	20–45 min	We can find patterns in when bilinguals decide which language to use. Bilinguals can choose to switch between both of their languages when they're with other bilinguals, but they will only use one language if they want to communicate with a monolingual.	Bilingual code‐switching
Categorical Perception	30–45 min	We can sort sounds by manner of articulation and by voicing, and read waveforms to determine whether the initial consonant is voiced or voiceless. If we use a computer to create a sound that is in between our categories, our brain still sorts it into a category.	Voicing, manner, and place of articulation; Interpreting graphs; Designing an experiment
Sound Illusions (McGurk & perceptual restoration)	10–15 min	Our brains combine all of the information available to us when listening to speech. Sometimes that is sounds + mouth movements, or sounds + what we already know about the world. When that information doesn't match, what we think we hear can be pretty funny!	Speech perception; Top‐down and bottom‐up processing
Speech Detectives	30–60 min	When people talk differently from us, we can usually find patterns and the differences are not random. We can find patterns in speech sounds by thinking about how the sounds are made.	Voicing, place, and manner of articulation; phonological patterns
Stroop (How powerful is your brain?)	5–15 min	Once we learn how to read, it becomes an automatic process. We are experts at reading in our native language(s).	Interference; Language processing
What is a Language?	45 min	Animals like honeybees communicate in different ways, but there are unique features of human languages.	Design features of language (Hockett, 1960)

### 4.1 Case study: Speech Detectives

Our *Speech Detectives* activity was originally created by a speech‐language pathologist to guide students through identifying the patterns in the speech of a child with a phonological disorder. Participants learn to describe speech sounds by place, manner, and voicing, and use those categories to identify patterns in how sounds change. Students see only a few words at a time to encourage them to repeatedly produce a hypothesis to explain the patterns in the data and then update that hypothesis when new data are added.

The activity originated as a worksheet that we used for in‐person activities at science fairs and visits to high school classes. To guide participants towards identifying the pattern of final consonant devoicing, participants see a set of target words ending with the /z/ sound and corresponding error pronunciations ending with the /s/ sound. Participants make a hypothesis and quickly identify that the /z/ sound changes to an /s/. The next set of data shows target words with an initial or medial /z/ sound next to the correct pronunciations, and participants revise their hypothesis to state that the /z/ sound becomes a /s/ sound only at the ends of words. Next, participants see words ending in /b/ and /d/ with corresponding error pronunciations ending in /p/ or /t/, respectively. At this point, most participants suggest three separate patterns: /b/ ‐> /p/, /d/ ‐> /t/, and /z/ > /s/ at the end of words.

Finally, the facilitator invites problem‐solving by asking the participants to find one pattern that describes all three patterns. We start by focusing on the differences between /s/ and /z/. Participants say both sounds out loud and share how the sounds are similar and how they are different. Children as young as second grade have told us that their mouth does the same thing for both and describe the voicing of /z/ as their tongue moving or as ‘vibration’. We give brief instruction about the larynx and vibration and show students how to feel their throats while saying sounds to determine if the sound is voiced or voiceless. After categorizing consonant sounds as voiced or voiceless, students identify that voiced consonants become voiceless at the ends of words in our data set. To wrap up the activity, students practice applying the pattern of final consonant devoicing to a new set of words.

**FIGURE 2:**
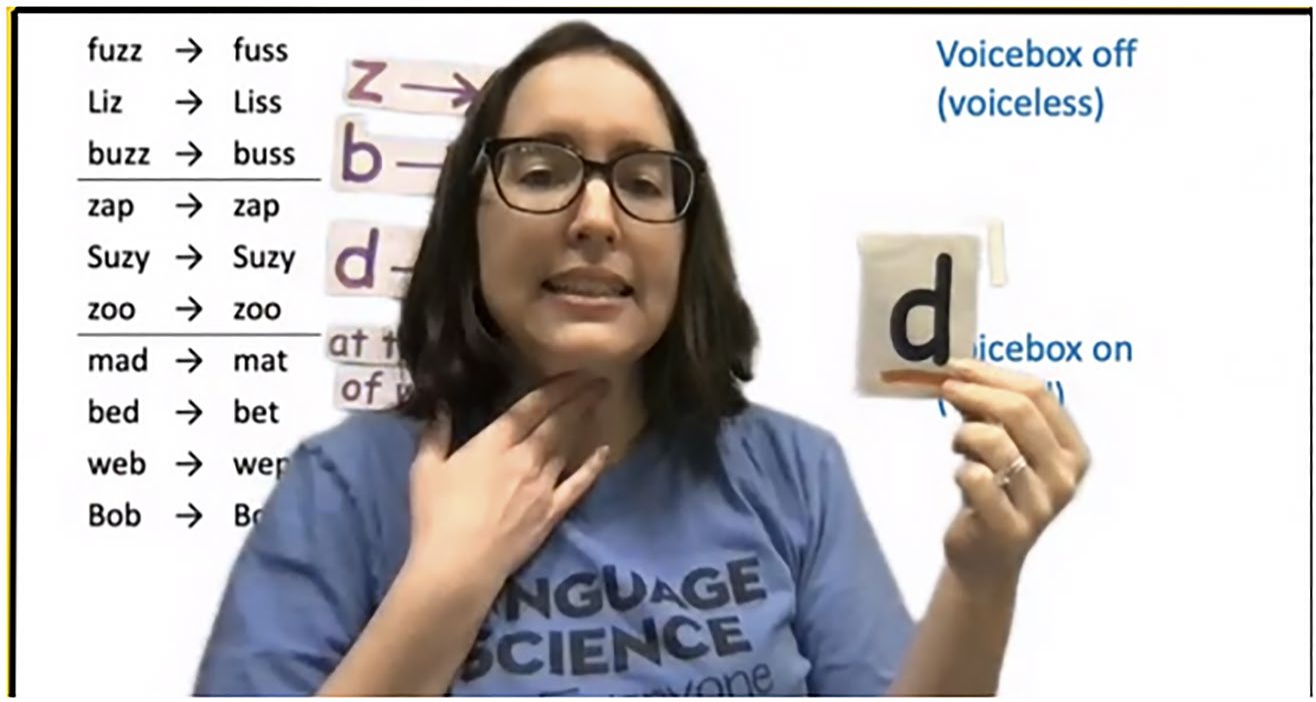
One of the authors sorts sounds with students during the *Speech Detectives* activity

To adapt this activity to be a virtual demonstration, we adjusted the list of words used in the data sets so that they could all be represented with an image. Using pictures makes the activity more engaging and reduces the focus on print. We use a drawing of a boy wearing a mask as the child with a phonological disorder. For each word in the data set, a speech bubble appears next to him containing a picture of the target word, and the child's pronunciation appears over the image. A running list of words and pronunciations is maintained as the slides advance. As students make hypotheses, felt pieces containing patterns such as ‘z ‐> s’ are placed on the green screen. We also use printed letters mounted on felt when sorting sounds into categories (Figure 2).

Students in grades 2 through 12 find this activity engaging and are able to solve the puzzle, coming away with a deeper understanding of the ways that one can study the sounds of language. To adapt the presentation for different ages, we introduce it to elementary school students by saying that the child pronounces some words differently than the facilitator, and our job is to figure out the pattern. For high school students, we say that they are going to be speech‐language pathologists and diagnose the boy's speech disorder. We find that regardless of age, students ask similar questions at the end about whether the boy has an accent or a disorder and how we can tell the difference, and such questions lead to meaningful discussions about multilingualism, descriptivism, and communication disorders. With our emphasis on developing, testing, and revising hypotheses, students are not only exposed to the study of language as a science, but they also learn that forming an incorrect hypothesis is a common experience in science. We hope that this emphasis on science as a process will help students see science as a method of problem‐solving rather than rote knowledge or procedures.

### 4.2 Case study: Bilingual Barnyard

The *Bilingual Barnyard* activity—created by a different group of graduate students at our university several years ago—centres around animal onomatopoeia and was designed to teach students about the sounds of languages around the world. In this demonstration, students attempt to guess the animals inside four barns after speakers of different languages say their onomatopoeic word for the sound that animal makes.

**FIGURE 3:**
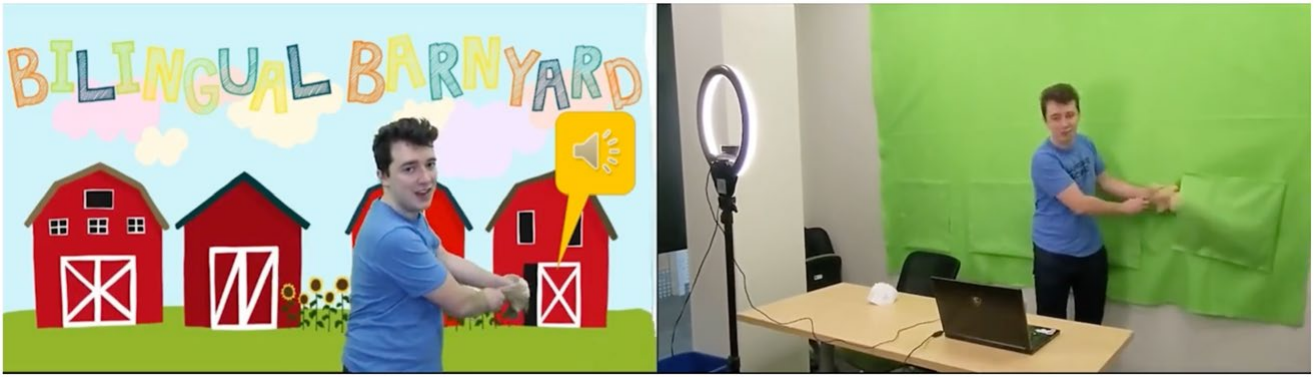
One of the authors pulls a dog puppet from a barn during the *Bilingual Barnyard* activity. The image on the left shows the audience view from Zoom, and the image on the right shows the green screen setup. Artwork credit: Mina Herzel

Originally, a presenter would walk a student through an activity on a tablet at a booth during a science fair or STEM night. One key factor of engagement in the in‐person version of this activity is its interactivity, where the students themselves select each barn and the activity evolves as a game. To keep this interactivity when moving this activity online, we leverage green screen technology and engaging visuals. In the online version, the presenter stands in front of the barns and interacts with the background by ‘knocking’ on the barn that students select to ‘wake up’ the animal inside. After the animal sounds play and the students make their guesses, the experimenter reveals a puppet of the animal inside the barn by pulling it from a green felt pocket attached to the green screen at the location of the barn, giving the appearance that the animal is emerging from the barn (Figure 3). The presenter then shows an image of the animal with speech bubbles containing the onomatopoeia that the students heard. We maintain the active student involvement from the in‐person activity as students can select the barns in any order. This new version also allows us to reach many students simultaneously, rather than just one student at a time. We solicit responses from the group of students, and they can raise their hands to agree with their peers, maintaining the game‐like presentation of the activity to a wide audience.

Having children with us for a full class period has also allowed us to extend the demonstration to move students from guessing answers in the first part of the activity to making predictions based on observable patterns. After playing the barn guessing game, we teach students some basic information about the syllable structure of English, specifically what sounds English syllables start or end with, and ask students to evaluate whether various sound sequences fit the patterns of English. We then tell students that not every language has the same patterns, and we describe basic syllable constraints of German and Japanese. Finally, students hear several examples of animal onomatopoeia in Japanese and German and deduce what language each word comes from by identifying which language′s patterns are consistent with the syllable structure they heard. This extended activity provides a more enriching experience for students as it allows us to teach them some specific examples of how the phonology of various languages differs and engage them in problem‐solving.

To address misconceptions throughout both parts of *Bilingual Barnyard*, we avoid wording which students could misinterpret to take away unintended prejudices. For example, we are careful to say that languages ‘do/don't’ have certain sounds, rather than that speakers ‘can/can't’ say specific sounds, to be clear that we are not laying out specific rules of what different speakers are allowed to say nor making value judgements about the features of the languages themselves.

## 5 IMPACT

**FIGURE 4:**
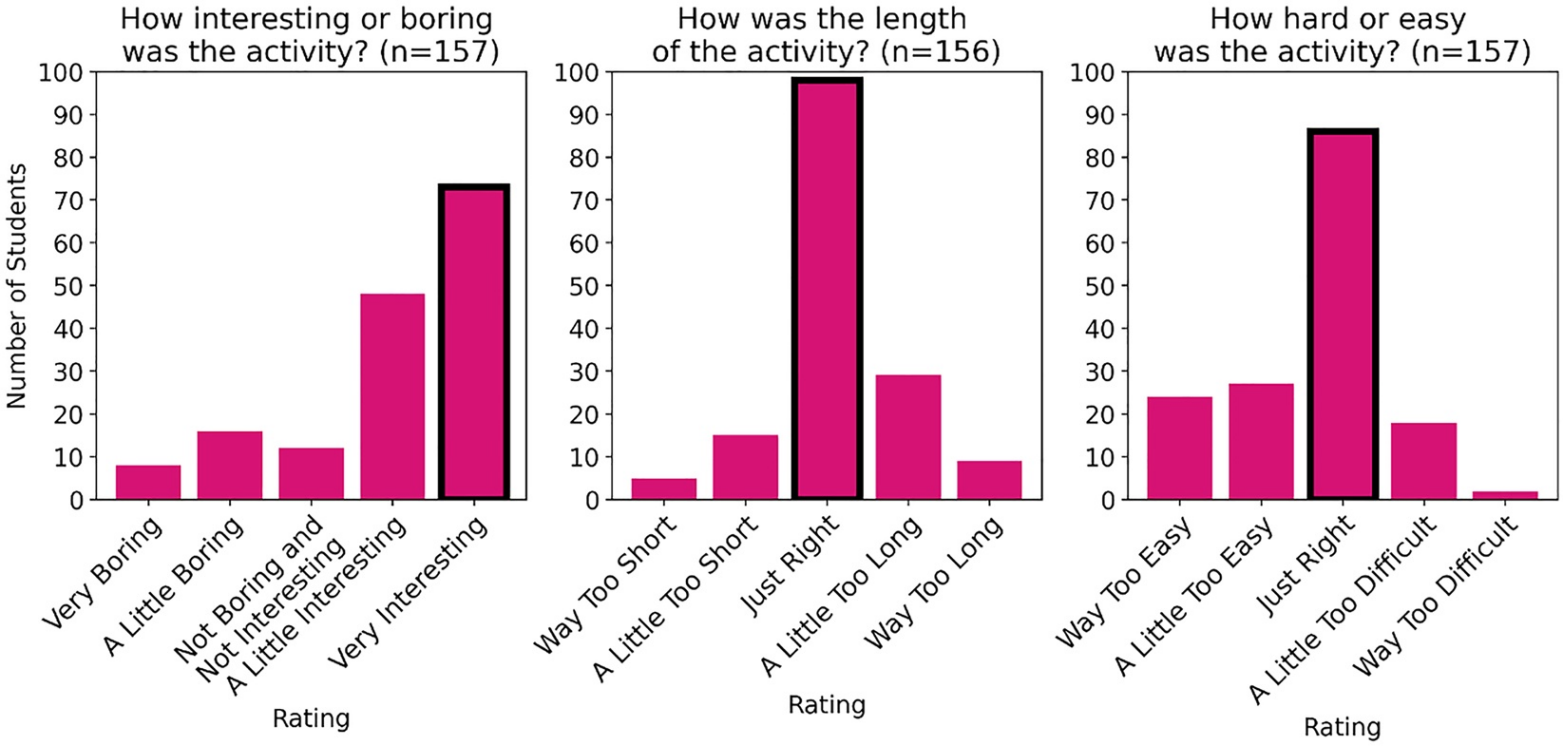
Distribution of survey responses from 157 s, third, and fourth graders after participating in a virtual language science outreach activity. For each question, the modal response (dark outline for emphasis) was the one that represented the ‘best’ rating, suggesting that overall our presentations were successful.

Our innovative approach to virtual language science outreach has several clear benefits to students, teachers, and the wider community. For students, the approach provides richer content and a deeper dive into language science as a field than short‐form activities. We hope that this provides a greater appreciation for language science and a strong introduction to a field that students may not typically experience until high school or later. To evaluate our impact on students, we administer a short survey that is formatted as a worksheet after each presentation. The worksheet and research have been approved by our university′s Institutional Review Board (IRB) for human subjects research. So far, we have received responses from 157 students in grades 2 through 4, approximately corresponding to ages 7–10. Students saw either a presentation of the *Bilingual Barnyard* or *Speech Detectives* demonstrations explained above, or our *Bilingual Code‐Switching* activity (see Table 1). Most students found our activities engaging—with 75% rating the activity as either ‘a little interesting’ or ‘very interesting’. Also, most students rated the activity as the right level of difficulty (53%) and the right length (60%). A distribution of responses is shown in Figure 4.

An in‐depth analysis of qualitative responses from students about what they learned is beyond the scope of this paper; however, student comments such as *‘I felt really happy that I got to learn about making hypothesis in how* [character in an activity] *speaks’* and *‘I learned that a hypothesis is an educated guess that you can test on’* suggest that our methods are effective in teaching students about forming hypotheses about the scientific study of language.

Teachers have incorporated our presentations into the curriculum in a variety of ways. In one recent collaboration, the teachers viewed our activities as part of the school's diversity and inclusion efforts for our focus on variation between languages while affirming that all languages are valid. In other schools, our activities are a part of a foreign language learning curriculum or part of the general STEM curriculum to show students examples of developing and testing hypotheses. While we aspire to collaborate with teachers as recommended in Lidz and Kronrod (2014), we have found that during the pandemic teachers prefer us to come with prepared presentations. We hope to collaborate with teachers in the future to further integrate our activities with school curricula.

The engagement of students in language science at an early age also has benefits for the wider language science community. We hope that introducing young students to this field may lead to wider knowledge of related fields when students are considering careers and undergraduate studies (Pearson et al., 2015). Parents and teachers are also not typically familiar with the concepts we present, so our activities are educational for adult audience members, too. Our goal of addressing misconceptions at a young age also has implications for the field more broadly, where much work has been spent trying to achieve the same goal with adults (e.g., Wolfram, 2010). By targeting children with our outreach and taking care to avoid reinforcing misconceptions, we may be able to preemptively limit such stereotypes from forming. Children's responses to our survey suggest that they take away descriptivist messages from our presentations: *‘…language scientists study all types of peoples languages that they speak’, ‘I learned that some people used different languages to other people so they can understand it’, ‘It's cool how* [character in an activity] *can speak two languages’.*

## 6 WHAT WE LEARNED

### 6.1 About teaching language science

In addition to benefiting the students and teachers we visit, this method of outreach also helps us as presenters. We improve our presentation skills and practice communicating our work to broader audiences in a way that we find enjoyable and rewarding and that fulfils outreach requirements from several funding agencies. The longer demonstrations require a more intimate knowledge of the source material, so we tend to create or lead demonstrations somewhat related to our own expertise which provides an opportunity to think more deeply about how to teach the material. The virtual format also emphasizes the value of guided discovery and the importance of making our presentations engaging when teaching new content, which are principles that apply to more than just doing outreach with elementary school students. When working with adult learners, as well, it can be helpful to engage with the scientific process and hypothesis development for topics that are relatively basic in the field, to avoid misconceptions, and demonstrate the methods we use to arrive at scientific knowledge (Bromme & Goldman, 2014; Loyens et al., 2015). These are skills that we can continue to use in our teaching careers, such as when teaching introductory classes to undergraduates and giving virtual posters and presentations for academic audiences.

### 6.2 About conducting research in schools

We also gained skills in working with teachers and conducting research in schools through arranging these visits and gathering our reflection surveys at the end of our presentations. In particular, this experience has made clear the importance of flexibility and clear communication when coordinating with schools and teachers (Reaser & Adger, 2007). Individual teachers arrange technology within their classrooms in different ways and have different expectations regarding how students interact with guest speakers; our role is to adjust our methods to accommodate those preferences, while still accomplishing our goals. Relatedly, we found that teachers varied in how they prefer their students to fill out our post‐activity reflection survey (e.g., paper forms vs. on individual laptops) and that we had to include a variety of survey distribution methods in the research protocol approved by our university's IRB.

## 7 CONCLUSION

In this article, we have discussed a method of doing virtual language science outreach activities with elementary school students that allows them to experience how scientific principles can be applied to questions about language. Four guiding principles drive our work: inviting problem solving, simplifying terms, addressing misconceptions, and encouraging interaction. These guiding principles help to ensure that students are receiving our intended takeaway messages, by reminding us to focus on being accurate and unbiased yet accessible to somebody with limited prior knowledge, and to provide many opportunities to interact and be engaged in the activities. Scientists doing other forms of outreach may find these guiding principles useful in their own work as well. Reactions from 157 students who have participated in our activities suggest that they are an appropriate length and level of difficulty. By focusing on encouraging interaction and live engagement even over Zoom and by using principles of guided discovery, we successfully taught elementary school students about studying language as a science in a way that they found enjoyable.

### 7.1 Limitations to this style of outreach

Though conducting our language science outreach through these longer‐form virtual activities has clear benefits for us as language scientists and for the children and teachers whose classrooms we visit, there are limitations to doing our outreach in this way that we did not face when focusing on shorter booth‐style interactions. Because most of our activities last at least 20 min and involve discovering a concept through student interaction, the scientist who is presenting the activity needs to have a high degree of familiarity with the subject as well as skill in leading young learners in discovery‐based learning. Presenters must be able to respond to unexpected questions or comments from students in a way that positively addresses their contribution to the activity without losing sight of the activity's goal, which requires more practice and expertise than managing short, one‐on‐one interactions. Additionally, creating a new activity involves multiple drafts to fine‐tune the outline, visuals, and take‐away message, which requires an extensive time commitment.

Additionally, there are some technological limitations to this method of outreach. We are only able to visit classrooms that have the technology to project a Zoom call onto a large screen and microphones to facilitate interaction. However, being virtual allows us to reach classrooms located farther from us that might not otherwise have access to university‐based linguists, and we expect to continue to reach students remotely after Covid‐19 restrictions are fully lifted. Many school buildings upgraded their technology to prepare for hybrid instruction due to Covid‐19, including screens and microphones in the classroom, making this method more feasible for many teachers. While we expect that our lessons learned from virtual outreach can be carried over when returning to in‐person guest speaker events, we are hopeful that technology will allow us to continue to reach diverse student groups even as pandemic restrictions are lifted.

### 7.2 Future plans

We encourage other language scientists to replicate and build on the work that we and others have been doing to bring language science to elementary school students. Language scientists whose outreach work has a different focus—such as increasing awareness of language diversity with the general public or showing high schoolers a different future career path—may find that some of our methods for encouraging interactivity also support their goals. We are continuing to create new demonstrations and to collect data from students regarding what they learn from our activities. Our hope is that gathering this information about the effectiveness of our outreach will help us as a field focus our time on activities that are most likely to support our goals. Though different outreach goals, audiences, settings, and resource restrictions will necessitate different types of activities, an evidence base regarding what children take away from each activity may help provide a framework for others becoming involved in language science outreach. We also plan to further analyse the written responses collected from students about what they learned in each activity to assess the distribution of topics that students learn about and find most interesting (e.g., developing hypotheses, studying multiple languages, learning about how different people talk, etc.). Additionally, we hope to work more closely with teachers in the future so that our presentations can be integrated into classroom instruction (Lidz & Kronrod, 2014).

## AUTHOR CONTRIBUTION

The authors made the following contributions to the paper: **Kathleen Oppenheimer**: Conceptualization; Methodology; Investigation; Writing – original draft; Writing – review & editing; Supervision, Project Administration. **Lauren Salig**: Methodology; Investigation; Writing – original draft; Writing – review & editing. **Craig Thorburn**: Methodology; Investigation; Visualization; Writing – original draft; Writing – review & editing. **Erika Exton**: Methodology; Investigation; Writing – original draft; Writing – review & editing.
